# Poster Session II - A219 BREASTFEEDING PRACTICES IN MOTHERS WITH INFLAMMATORY BOWEL DISEASE: A PROSPECTIVE COHORT STUDY

**DOI:** 10.1093/jcag/gwaf042.219

**Published:** 2026-02-13

**Authors:** S Chen, K O’ Connor, V Srikanth, G Nguyen, C Maxwell, V W Huang

**Affiliations:** University of Toronto Temerty Faculty of Medicine, Toronto, ON, Canada; Department of Gastroenterology, Mount Sinai Hospital, Toronto, ON, Canada; Department of Gastroenterology, Mount Sinai Hospital, Toronto, ON, Canada; Department of Gastroenterology, Mount Sinai Hospital, Toronto, ON, Canada; Obstetrics and Gynecology, Mount Sinai Hospital, Toronto, ON, Canada; Department of Gastroenterology, Mount Sinai Hospital, Toronto, ON, Canada

## Abstract

**Background:**

Inflammatory bowel disease (IBD), which includes Crohn’s disease (CD) and ulcerative colitis (UC), introduces challenges for breastfeeding such as concerns over infant medication exposure. Studies suggest that IBD mothers may discontinue breastfeeding earlier than healthy mothers though overall, breastfeeding patterns in women with IBD remain poorly studied.

**Aims:**

To further investigate the factors influencing breastfeeding practices in women with IBD.

**Methods:**

Women with IBD were enrolled in the Preconception and Pregnancy in IBD Prospective Cohort study at the Mount Sinai Hospital (Toronto, ON) starting in 2018. Those who had delivered an infant completed postpartum surveys from delivery to 12 months postpartum. These surveys included breastfeeding habits (e.g. reasons for discontinuation, if applicable), mental health, disease activity, medication use, and IBD-specific pregnancy knowledge (using the Crohn’s and Colitis Pregnancy Knowledge Score). Breastfeeding rates were compared between CD and UC groups, with statistical analyses including chi-square tests and Fisher’s Exact tests.

**Results:**

A total of 111 IBD mothers were included (58 with CD, 53 with UC). Breastfeeding rates at delivery (99%), and 2 months (96%) was high, but declined at 4 months (77%) and then plateaued (77% at 6 months, 73% at 8 months, and 72% at 12 months). The largest drop in breastfeeding occurred at 4 months, primarily due to concerns over insufficient milk supply (16%) and concern of IBD medication use (5%). At this time, 30% of mothers who stopped breastfeeding had active disease.

Anxiety did not affect breastfeeding rates at 2 months (Fisher’s Exact, p > 0.05). All mothers with minimal or mild anxiety breastfed, compared to 67% of those with moderate anxiety. By 12 months, mothers with minimal or moderate anxiety were more likely to continue breastfeeding compared to those with mild or severe anxiety. Across all postpartum timepoints, biologics were the most common therapy, and breastfeeding rates did not vary across medication class.

**Conclusions:**

Most mothers with IBD initiate breastfeeding, but rates decline at four months postpartum due to medication safety concerns and breastfeeding challenges. Early postpartum anxiety did not impact breastfeeding initiation; however, by 12 months, mothers with mild or severe anxiety were less likely to continue breastfeeding than those with minimal or moderate anxiety. This may reflect cumulative stress or prior anxiety symptoms rather than anxiety at a single time point, highlighting the need for further study.

**Funding Agencies:**

CAN TAP TALENT Summer Student Award

